# SELECTIVE DORSAL RHIZOTOMY IN CEREBRAL PALSY: SELECTION CRITERIA AND POSTOPERATIVE PHYSICAL THERAPY PROTOCOLS

**DOI:** 10.1590/1984-0462/;2018;36;1;00005

**Published:** 2018-01-15

**Authors:** Renata D’Agostini Nicolini-Panisson, Ana Paula Tedesco, Maira Rech Folle, Márcio Vinicius Fagundes Donadio

**Affiliations:** a Pontifícia Universidade Católica do Rio Grande do Sul, Porto Alegre, RS, Brasil.; b Instituto de Neuro-Ortopedia, Caxias do Sul, RS, Brasil.

**Keywords:** Muscle spasticity, Rhizotomy, Physical therapy specialty, Postoperative care, Cerebral palsy, Espasticidade muscular, Rizotomia, Fisioterapia, Cuidados pós-operatórios, Paralisia cerebral

## Abstract

**Objective:**

To identify selection criteria for selective dorsal rhizotomy (SDR) in cerebral palsy, to analyze the instruments used for evaluation, and to describe the characteristics of physical therapy in postoperative protocols.

**Data sources:**

Integrative review performed in the following databases: SciELO, PEDro, Cochrane Library, and PubMed. The terms in both Portuguese and English for “cerebral palsy”, “selective dorsal rhizotomy”, and “physical therapy” were used in the search. Studies whose samples enrolled individuals with cerebral palsy who had attended physical therapy sessions for selective dorsal rhizotomy according to protocols and describing such protocols’ characteristics were included. Literature reviews were excluded and there was no restriction as to period of publication.

**Data synthesis:**

Eighteen papers were selected, most of them being prospective cohort studies with eight-month to ten-year follow-ups. In most studies, the instruments of assessment encompassed the domains of functions, body structure, and activity. The percentage of posterior root sections was close to 50%. Primary indications for SDR included ambulatory spastic diplegia, presence of spasticity that interfered with mobility, good strength of lower limbs and trunk muscles, no musculoskeletal deformities, dystonia, ataxia or athetosis, and good cognitive function. Postoperative physical therapy is part of SDR treatment protocols and should be intensive and specific, being given special emphasis in the first year.

**Conclusions:**

The studies underline the importance of appropriate patient selection to obatin success in the SDR. Postoperative physical therapy should be intensive and long-term, and must necessarily include strategies to modify the patient’s former motor pattern.

## RESUMO

**Objetivo:**

Identificar critérios de seleção para a rizotomia dorsal seletiva (RDS) na paralisia cerebral (PC), analisar os instrumentos de avaliação e descrever as características da fisioterapia nos protocolos pós-operatórios.

**Fontes de dados:**

Revisão do tipo integrativa nas bases de dados SciELO, PEDro, Cochrane Library e PubMed. Os termos em português e inglês “paralisia cerebral”, “rizotomia dorsal seletiva” e “fisioterapia” foram utilizados na busca. Os critérios de inclusão foram: artigos que arrolaram indivíduos com PC, que realizaram fisioterapia nos protocolos de RDS e que descreviam características desses protocolos. Foram excluídos artigos de revisão da literatura e não houve restrição de período de publicação.

**Síntese dos dados:**

Incluíram-se 18 estudos, sendo a maioria coortes prospectivas, com acompanhamento dos pacientes de oito meses a dez anos. Os instrumentos das avaliações contemplam, na maior parte dos trabalhos, os domínios de funções e estruturas corporais e atividade. O percentual de secção das raízes posteriores foi próximo a 50%. A principal indicação para a RDS incluiu deambuladores com diplegia espástica, que preenchiam os seguintes critérios: espasticidade que interfere com a mobilidade, boa força muscular de membros inferiores e tronco, sem deformidades ortopédicas, distonia, ataxia ou atetose e boa função cognitiva. A fisioterapia é parte integrante dos protocolos de tratamento com RDS, devendo ser intensiva, específica e enfatizada principalmente no primeiro ano.

**Conclusões:**

Os estudos salientam a importância da seleção adequada dos pacientes para o sucesso dos resultados. A fisioterapia é intensiva e de longa duração, devendo necessariamente ter estratégias para modificar o antigo padrão motor.

## INTRODUCTION

Spasticity is the main clinical feature of patients with spastic cerebral palsy (CP) and is considered the most important cause of discomfort, gait abnormalities, and functional limitations.^1^ It also generates muscle shortenings that influence bone growth and lead to deformities. Controlling it, therefore, is crucial to the treatment of CP.^2^

Selective dorsal rhizotomy (SDR) is a neurosurgical procedure performed in children with bilateral spastic CP to reduce lower limb spasticity.^3^ It is mostly performed at the lumbosacral level and consists of the interruption of the afferent stimulus by the monosynaptic stretch reflex.^3^ In order to preserve the sensory and sphincter functions, the dorsal root is divided into radicles and only a portion of these is sectioned.^3^

SDR was first described by Foerster in 1908, after he observed that the dorsal (sensory) radicles section could decrease spasticity; significant muscle weakness with sensory and proprioceptive losses was also observed after the procedure.^2^ Thus, in 1978, Fasano presented the intraoperative electrophysiological stimulation and the section of a portion of dorsal radicles, and both techniques are currently used.^2^ The method was then adopted and popularized by Peacock and Arens in 1980.^2^

SDR results indicate spasticity reduction, muscle strength gain, gait speed and kinematics increase, and gross motor function improvement.^4^ ^,^ ^5^ ^,^ ^6^Patients submitted to SDR and physical therapy are compared to with those who only received physical therapy, significant reduction in spasticity and functional improvement are seen in the first group.^7^ ^,^ ^8^ Specific physical therapy plays a central role in the postoperative phase, as spinal bone procedures such as laminectomy or laminotomy require special care in the first weeks of this period, in addition to formal conduct.^9^ ^,^ ^10^

The centers that offer SDR follow special protocols for the postoperative period. In Brazil, this technique is starting to be disseminated and, due to peculiarities related to postoperative treatment, this review of protocols described in the literature aims to help professionals to better understand the role of physical therapy in rehabilitation. The objectives of this study were, therefore, to identify SDR selection criteria and to describe the characteristics of physical therapy postoperative protocols.

## DATA SOURCE

This is an integrative literature review. The electronic search for references was carried out in August 2016 in the databases SciELO, PEDro, Cochrane Library, and PubMed. The terms used in the search, both in Portuguese and in English, were: “*paralisia cerebral”*/“cerebral palsy”, “*rizotomia dorsal seletiva”*/“dorsal selective rhizotomy”, and “*fisioterapia”*/“physical therapy”. Headings, abstracts and, when necessary, the full study were reviewed to determine whether they would match inclusion criteria: studies conducted with individuals with CP who had attended physical therapy sessions according to SDR protocols and depicting such protocols’ characteristics. No filters were applied to search, as well as there were no restrictions as to age group of sample subjects or period of publication. Literature reviews were excluded. The lists of references of selected papers were also searched for other relevant manuscripts.

After selection, the authors made a critical reading to grasp the main information, which was then presented in the following categories:

- characteristics of studies;
- characteristics of study samples.
- SDR selection criteria;
- characteristics of physical therapy protocols.

## DATA SYNTHESIS

**Figure 1:**
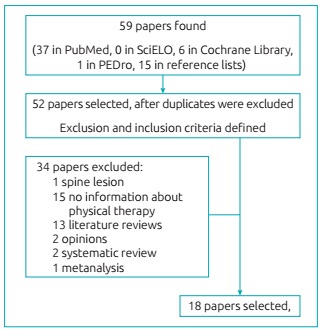
Flowchart showing the search and selection of papers.

According to the pre-established criteria, 18 articles were selected for this review. Figure 1 is the flowchart of papers’ search and selection.

### Characteristics of studies 

The studies included in our research are shown in Table 1. From 18 studies included, five (27.8%) were randomized clinical trials,^6^ ^,^ ^8^ ^,^ ^11^ ^,^ ^12^ ^,^ ^13^six (33.3%) were series of cases (four prospective^14^ ^,^ ^15^ ^,^ ^16^ ^,^ ^17^ and two retrospective^4^ ^,^ ^7^), and seven (38.9%) were prospective cohort studies.^5^ ^,^ ^18^ ^,^ ^19^ ^,^ ^20^ ^,^ ^21^ ^,^ ^22^ ^,^ ^23^ Sample sizes ranged from 9 to 68 individuals, most of them being distributed in groups of SDR intervention associated with physical therapy^4-^ ^8^ ^,^ ^11^ ^,^ ^13^ ^,^ ^14^ ^,^ ^16^ ^,^ ^17^ ^,^ ^18^ ^,^ ^19^ ^,^ ^21^ ^,^ ^22^ ^,^ ^23^or only physical therapy.^4^ ^,^ ^6^ ^,^ ^8^ ^,^ ^11^ Patient follow-up periods ranged from eight months^15^ to ten years.^22^ ^,^ ^23^ In most studies, the same physical therapist performed both pre- and postoperative evaluations.

**Table 1:** Characteristics of papers included in the review.

Study	Design	n	Groups	Evaluations	ICF
Graubert et al.^6^	Blinded randomized controlled trial	32	SDR + PT PF	Basal, 6, 12 and 24 months	BF/BS, A
Wright et al.^12^	Randomized clinical trial	24	SDR + PT + OT PT + OT	Basal, 6 and 12 months	BF/BS, A
McLaughlin et al.^14^	Prospective case series	34	SDR + PT	Basal and ±12 months (10-18)	BF/BS, A
McLaughlin et al.^11^	Blinded randomized controlled trial	38	SDR + PT PT	Basal 6, 12 and 24 months	BF/BS, A
Josenby et al.^22^	Prospective cohort	29	SDR + PT	Basal, 6, 12 and 18 months; 3, 5 e 10 years	BF/BS, A
Chan et al.^7^	Retrospective case series	22	SDR + PT	Basal, 2 weeks; 3, 6 and 12 months	BF/BS, A, P
Engsberg et al.^18^	Prospective cohort	22	SDR + PT	Basal, 2 years	BF/BS, A
Engsberg et al.^4^	Retrospective case series	68	SDR + PT PT ND	Basal, 8 and 20 months	BF/BS, A
Schie et al.^16^	Prospective case series	9	SDR + PT	Pre-SDR: mensal (4 months); Post-SDR: bimensal (12 months)	BF/BS, A, P
Engsberg et al.^17^	Prospective case series	59	SDR + PT	Basal, 8 and 24 months	BF/BS
Buckon et al.^21^	Prospective cohort	18	SDR + PT	Basal, 6 and 12 months	BF/BS
Steinbok et al.^13^	Randomized clinical trial	26	SDR + PT PT + SDR + PT	Basal, 9 and 18 months	BF/BS, A
Engsberg et al.^15^	Prospective case series	25	SDR + PT + HEP PT + HEP	Basal, 8 months	BF/BS, A
Hodgkinson et al.^19^	Prospective cohort	18	SDR + PT	3 months (pre-SDR), 1, 2 and 3 years	BF/BS, A
Dudgeon et al.^20^	Prospective cohort	29	SDR + OT + PT	Basal, 6 and 12 months	BF/BS, A, P
Josenby et al.^23^	Prospective cohort	24	SDR + PT	Basal, 6, 12 and 18 months, 3, 5 and 10 years	A, P and PF
Nordmark et al.^5^	Prospective cohort	35	SDR + PT	Basal, 6, 12 and 18 months, 3 and 5 years	BF/BS, A, P
Steinbok et al.^8^	Blinded randomized controlled trial	28	SDR + PT PT	Basal, 3, 6 and 9 months	BF/BS, A, P

n: sample size; SDR: selective dorsal rhizotomy; PT: physical therapy; ND: no disability; OT: occupational therapy; HEP: home exercise program; ICF: International Classification of Functioning, Disability and Health*;* BF/BS: body function, body structure; A: activity; P: participation; PF: personal factors.

### Evaluation tools according to domains of the International Classification of Functioning, Disability and Health

The evaluation of SDR candidates should be as comprehensive as possible and encompass elements described by the International Classification of Functioning, Disability and Health (ICF). One of the studies^7^ used the quantitative classification by ICF, and another one made evaluations considering ICF domains, as described below.

The literature brings a variety of information with respect to items to be evaluated, with domain, structure, and body function as per ICF considered in all studies but one.^23^ The instruments used in studies to evaluate domain, structure, and body function were: spasticity evaluation (Ashworth scale,^5^ ^,^ ^6^ ^,^ ^7^ ^,^ ^8^ ^,^ ^11^ ^,^ ^12^ ^,^ ^13^ ^,^ ^14^ ^,^ ^16^ ^,^ ^22^ clinical signs of spasticity,^11^ ^,^ ^15^ quantitative spasticity assessment (QSA)^6^ ^,^ ^11^ ^,^ ^19^ by isokinetic dynamometer^4^ ^,^ ^17^ ^,^ ^18^), motion range,^5^ ^,^ ^6^ ^,^ ^7^ ^,^ ^8^ ^,^ ^11^ ^,^ ^12^ ^,^ ^13^ ^,^ ^14^ ^,^ ^15^ ^,^ ^19^ ^,^ ^22^, reflex range,^12^ ^,^ ^14^ muscle strength,^4^ ^,^ ^8^ ^,^ ^13^ ^,^ ^15^ ^,^ ^17^ ^,^ ^18^ ^,^ ^19^, popliteal angle,^22^ musculoskeletal deformities^14^ by hips and spine radiography,^7^ selectivity assessment^7^, and isometric contraction assessment by electromyography.^21^

Only two studies^17^ ^,^ ^21^ did not measure the activity domain, and in those addressing it, the instruments used were: Gross Motor Function Measure (GMFM),^4^ ^,^ ^5^ ^,^ ^6^ ^,^ ^7^ ^,^ ^8^ ^,^ ^11^ ^,^ ^12^ ^,^ ^14^ ^,^ ^16^ ^,^ ^18^ ^,^ ^22^ walking status^6^ ^,^ ^8^ ^,^ ^11^ ^,^ ^14^, Gross Motor Classification System (GMFCS),^5^ ^,^ ^7^ ^,^ ^22^ ^,^ ^23^ three-dimensional gait analysis,^4^ ^,^ ^6^ ^,^ ^7^ ^,^ ^12^ ^,^ ^18^ observational gait analysis^7^ ^,^ ^16^ (Observational Gait Score^7^, Edinburgh Visual Gait Score^16^), urodynamics,^7^ Peabody Fine Motors Scale,^8^ self-care evaluation,^8^ ^,^ ^20^ walking distance in 60 seconds^12^, and Physiological Cost Index.^8^

Six studies^5^ ^,^ ^7^ ^,^ ^8^ ^,^ ^16^ ^,^ ^20^ ^,^ ^23^ addressed the domain participation and its evaluation instruments: Pediatric Evaluation of Disability Inventory (PEDI),^5^ ^,^ ^7^ ^,^ ^16^ ^,^ ^20^ ^,^ ^23^ Canadian Occupational Performance Measure (COMP)^7^, and self-care evaluation.^8^ ^,^ ^20^ SDR evaluation should follow more comprehensive protocols with postoperative analysis of the same instruments, thus allowing a more accurate evaluation of results and better conclusion-drawing.

### Characteristics of study samples

Table 2 shows the characteristics of samples of the included studies. All of them enrolled individuals with spastic PC. The study by Chan et al mentioned a participant with hereditary spastic paraparesis (HSP), in addition to 20 individuals with CP.^7^ Although CP diagnosis was one of the inclusion criteria of this review, SDR can also be indicated for patients with spasticity resulting from other diagnoses such as multiple sclerosis, Leigh syndrome,^25^ stroke,^26^ spinal cord injury^24^, and transverse myelitis.^27^

**Table 2:** Characteristics of samples of papers included.

Study	Age	Topography	GMFCS	Level of section	Percentage of section	Surgical approach
Graubert et al.^6^	6.5 (3.3-14.5)^*^	Diplegia	-	-	-	-
Wright et al.^12^	58.0±12.7 (41-91) months	Diplegia	-	L2-S2	50	Partial laminectomy L2-L5
McLaughlin et al.^14^	QE: 7.2±3.4; DE: 8.9±3.9^**^	Diplegia, quadriplegia	-	L2-S2	49 (29-60)	Laminotomy T12-S2
McLaughlin et al.^11^	6.1±3.0 (2.9-14.3)^*^	Diplegia	-		34 (20-56)	Laminectomy or laminotomy
Josenby et al.^22^	4.3 (2.6-6.7)	Diplegia	I-V	-	-	-
Chan et al.^7^	8.6±2.6 (5.9-11.2)	Diplegia, quadriplegia	I-IV	L1-S2	49.7±2.2	Articulate laminotomy L2-S1
Engsberg et al.^18^	8.8±4.8	Diplegia	I-III	L1-S2	(60-65)	Laminotomy L1
Engsberg et al.^4^	9.0±5.3^*^	Diplegia	I-III	L1-S2	65	Laminotomy L1
Schie et al.^16^	65 (43-82) months	Diplegia	II-III	L2-S1	50 (31-68)	Laminotomy L1-L5
Engsberg et al.^17^	8.5±4.4 (4-18)^#^	Diplegia	I-III	L1-S2	-	Laminotomy L1-L2
Buckon et al.^21^	63 (48-86) months^#^	Diplegia	-	L2-S1	42 (36-48)	Laminotomy L2-L5
Steinbok et al.^13^	(3-7)	Diplegia	-	L2-S1	(33-62)	Laminotomy L1-S1
Engsberg et al.^15^	9±4.2 (4-16)^*^	Diplegia	-	L1-S2	(60-80)	Laminectomy L2 and, when needed, L1
Hodgkinson et al.^19^	9 (5.5-16.5)	Quadriplegia	-	-	60	Laminotomy T12-L2
Dudgeon et al.^20^	8.1±4.1 (3.7-22)	Diplegia, quadriplegia	-	L2-S1	42	-
Josenby et al.^23^	4.1 (2.5-6.4)	Diplegia	I-V	L2-S2	40	En-Bloc laminoplasty L1-L5
Nordmark et al.^5^	4.5±1.1 (2.5-6.6)	Diplegia	I-V	L2-S2	40	En-Bloc laminoplasty L1-L5
Steinbok et al.^8^	50 (35-75) months^#^	Diplegia	I-IV	L2-S2	45±5	Laminotomia L1-S1

Age: mean±standard deviation (min. and max. interval) shown in years, unless indicated otherwise; ^*^Group SDR + physical therapy; #Group CP; ^**^Group SQ: spastic quadriplegia; SD: spastic diplegia; GMFCS: Gross Motor Function Classification System; section percentage: mean±standard deviation (min. and max. interval); -- does not shown.

Only one study did not include individuals with CP due to spastic diplegia^19^ and four studies enrolled individuals with spastic quadriplegia.^7^ ^,^ ^14^ ^,^ ^19^ ^,^ ^20^ Regarding GMFCS levels, only half of the studies^4^ ^,^ ^5^ ^,^ ^7^ ^,^ ^8^ ^,^ ^16^ ^,^ ^17^ ^,^ ^18^ ^,^ ^22^ ^.^ ^23^ referred to this classification, and individuals presenting all levels are mentioned. Overall, SDR is the procedure of choice for spasticity of lower limbs in children with diplegia,^9^ since they have more involvement of the lower limbs and dystonia is not always present.^9^ Patients with spastic quadriplegia are more likely to present dystonia and involvement of both upper and lower limbs, and the treatment with continuous intrathecal baclofen infusion is more indicated,^9^ although some studies support SDR.^7^ ^,^ ^14^ ^,^ ^19^ ^,^ ^20^ Another aspect to be considered when indicating SDR is ambulation potential,^9^ which includes GMFCS levels I, II, and III. However, investigations have performed SDR for levels IV and V with specific goals and suggested that this is an alternative to the use of continuous intrathecal baclofen infusion, given the management and monitoring complexity of this method.^28^

Most studies had section of 50% of the selected posterior rootlets from L1 or L2 to S1 or S2. A meta-analysis showed direct relationship between the percentage of cut and function gain, that is, the decrease in spasticity helps in the acquisition of functional abilities.^1^

### SDR selection criteria 

As shown in Table 3, the studies usually have patients with spastic diplegia matching the selection criteria^4^ ^,^ ^6^ ^,^ ^7^ ^,^ ^11^ ^,^ ^12^ ^,^ ^13^ ^,^ ^14^ ^,^ ^15^ ^,^ ^16^ ^,^ ^17^ ^,^ ^18^ ^,^ ^20^ and the five “s”:^2^ ^,^ ^3^ ^,^ ^7^ spastic - lower limb spasticity interfering with functionality;^4^ ^,^ ^6^ ^,^ ^7^ ^,^ ^11^ ^,^ ^12^ ^,^ ^13^ ^,^ ^14^ ^,^ ^15^ ^,^ ^16^ ^,^ ^17^ ^,^ ^18^ ^,^ ^19^ ^,^ ^20^ ^,^ ^22^ strength - adequate lower limb muscle strength and control;^7^ ^,^ ^12^ ^,^ ^22^ straight - adequate trunk^6^ ^,^ ^7^ ^,^ ^22^ and head^6^ control without fixed orthopedic deformity;^7^ ^,^ ^11^ ^,^ ^12^ ^,^ ^16^ ^,^ ^17^ ^,^ ^22^ slim - being thin; and smart - not having significant cognitive deficits.^4^ ^,^ ^6^ ^,^ ^7^ ^,^ ^11^ ^,^ ^18^ Also, criteria including good family support are cited,^16^ ^,^ ^29^ as well as good rehabilitation^11^ ^,^ ^16^ and the capacity to collaborate in rehabilitation (cognitively and emotionally).^18^ Even though this is not the population to whom SDR is ideally indicated, some studies indicate it for patients with spastic quadriplegia^7^ ^,^ ^14^ ^,^ ^17^ ^,^ ^20^ with the following criteria:^3^ ^.^ ^7^ significant spasticity interfering with positioning, care, and passive stretching; absence of other motor disorders; and absence of fixed contractures in multiple joints. In both topographies, abnormalities of movement (dystonia, ataxia, choreoathetosis, hypotonia, stiffness),^4^ ^,^ ^6^ ^,^ ^7^ ^,^ ^11^ ^,^ ^13^ ^,^ ^17^ ^,^ ^18^ ^,^ ^22^ hips instability,^11^ significant scoliosis,^11^ presence of significant fixed contractures,^7^ ^,^ ^11^ ^,^ ^12^ ^,^ ^16^ ^,^ ^17^ ^,^ ^22^ absence of antigravity muscle strength,^11^ and visual impairments limiting mobility^11^ are contraindications for the procedure.

**Table 3:** SDR indication criteria in subjects with cerebral palsy.

Inclusion criteria	Exclusion criteria
3-18 years^6^ ^,^ ^11^ 3-21 years^14^ 3-7 years^8^ ^,^ ^13^ ^,^ ^16^ 4-17 years^15^ >2 years^17^ >4 years^4^ ^,^ ^18^ <7 years^5^ children, adolescents and young adult^20^	Bulbar involvement^6^ Dystonia, athetosis, rigidity, mild to severe hypotonia ^4^ ^,^ ^14^ ^,^ ^17^ ^,^ ^18^ ^,^ ^22^ ^,^ ^23^ Dystonia, athetosis, ataxia^5^ ^,^ ^7^ ^,^ ^8^ ^,^ ^11^ ^,^ ^13^ CNS malformation ^4^ ^,^ ^18^
Spastic diplegia^4^ ^,^ ^5^ ^,^ ^6^ ^,^ ^7^ ^,^ ^8^ ^,^ ^11^ ^,^ ^12^ ^,^ ^13^ ^,^ ^14^ ^,^ ^15^ ^,^ ^16^ ^,^ ^17^ ^,^ ^18^ ^,^ ^20^ ^,^ ^21^ Spastic quadriplegia with remarks^7^ ^,^ ^14^ ^,^ ^17^ ^,^ ^20^ ^,^ ^21^ ^,^ ^22^ ^,^ ^23^	Visual impairment limiting mobility^11^
Good head-trunk control^6^ ^,^ ^7^ ^,^ ^22^ ^,^ ^23^ LL reasonable muscle strength^12^ ^,^ ^22^ ^,^ ^23^	Depends on spasticity to stand up or walk^22^ ^,^ ^23^
Ability or potential to wander with and without supportive device,^4^ ^,^ ^6^ ^,^ ^12^ ^,^ ^18^ for three meters^12^ Able to walk barefoot for eight minutes, with or without support,^4^ ^.^ ^18^ to sit, kneel and crawl independently for short periods,^16^ to crouch seven times,^16^ sit on a bench with free arms and to stand up with support^8^ ^,^ ^13^	Fixed LL contractures^5^ ^,^ ^7^ Severe fixed contractures:^11^ ^,^ ^12^ ^,^ ^16^ ^,^ ^17^ ^,^ ^22^ ^,^ ^23^ >30º on hips and knee;^12^ >15º on hips and knee and >30º on ankle;^11^ >20º on hips, knee, and ankle and >80º popliteal angle
GMFCS I - III,^4^ ^,^ ^18^ I-V,^5^ II-III^16^	Progressive subluxation of the hips ^8^ ^,^ ^11^
36-month or more intellectual function^6^ ^,^ ^11^ Minimum cognitive skills to actively participate^4^ ^,^ ^5^ ^,^ ^18^ Children with intellectual disabilities^23^	Spinal deformities, uncontrolled epilepsy, contraindication for prolonged anesthesia^11^
Spasticity of LL interfering with functional tasks such as sitting, standing, and walking^4^ ^,^ ^5^ ^,^ ^7^ ^,^ ^8^ ^,^ ^12^ ^,^ ^13^ ^,^ ^14^ ^,^ ^16^ ^,^ ^17^ ^,^ ^18^ ^,^ ^19^ ^,^ ^20^ ^,^ ^22^ ^,^ ^23^ Spasticity in at least six muscle groups of both LL^16^	Orthopedic surgery,^4^ ^,^ ^12^ ^,^ ^18^ in the previous year^4^ ^,^ ^5^ ^,^ ^18^ or near-term planning^8^ Botulinum toxin or plaster in six months^4^ ^,^ ^18^
Availability for intensive physical therapy^5^ ^,^ ^8^ ^,^ ^11^ Good family and rehabilitation support^11^ ^,^ ^16^	Severe cognitive disability^4^ ^,^ ^5^ ^,^ ^7^ ^,^ ^11^ ^,^ ^18^

LL: lower limbs; GMFCS: Gross Motor Function Classification System; CNS: central nervous system.

The correct indication of SDR is fundamental for the success of treatment.^3^ ^,^ ^30^ Criteria have been described and the literature supports that it is important that this decision is made by a multidisciplinary team trained and specialized in the care of spasticity in CP patients and with access to all treatment options.^1^ ^,^ ^2^ ^,^ ^3^ ^,^ ^10^ ^,^ ^31^ This team should consist of a physical therapist, a pediatrician, an orthopedist, and a neurosurgeon, all of them trained and specialized.^1^ ^,^ ^31^ The whole staff, including patients’ family members, should agree with the SDR decision and with the individual treatment goals for each child.^2^ ^,^ ^9^ A recent systematic review stated that these selection criteria vary across studies and are based more on clinical reasoning than on scientific evidence, and it is important that specialists come to a consensus on the subject.^3^

### Characteristics of physical therapy protocols 

Table 4 lists the characteristics of post-SDR physical therapy protocols, including start of sessions, length of hospital stay and frequency. Studies typically show that, after SDR, patients undergo intensive physical therapy rehabilitation lasting approximately one year, starting on the first days after surgery and staying hospitalized from six days to six weeks. Two studies^13^ ^,^ ^15^ reported preoperative physical therapy and three^12^ ^,^ ^20^ ^,^ ^21^ mentioned postoperative occupational therapy as well.

**Table 4:** Characteristics of physical therapy protocols following selective dorsal rhizotomy.

Study	PT start (day)	Length of hospital stay	Physical therapy frequency
Graubert et al.^6^	--		4 weeks de terapia:10 hours/week + 5 months: 4-5 hours/ week + 6 months: 1-3 hours/week
Wright et al.^12^	2^nd^ or 3^rd^	6 weeks	6 weeks: 45 minutes/day of physical therapy e 2 sessions/week (45 minutes of occupational therapy); after discharge, up to 1 year: 2 sessions/week (120 minutes)
McLaughlin et al.^14^	4^th^ a 6^th^	1 month	1^st^ month: 2 hours/day for 5 days/week; following 5 months: 3-5 hours/week; 6^th^ month: normal therapy
McLaughlin et al.^11^	2^nd^	1 month	4 weeks: 2 hours/day for 5 days/week (40 hours) + 5 months:1 hour/day for 4-5 days/week + 6 months: 1 hour/day for 1-4 days/week
Josenby et al.^22^	1^st^	--	6 months: twice/week (1 hour); 6^th^-18^th^ month: once/week and physical activities
Chan et al.^7^	2^nd^	4 weeks	4 weeks: 5 hours/day for 5 times/week; 2^nd^ -12^th^ month: 3-6 hours/week
Engsberg et al.^18^	5^th^	1 week	5^th^ day-8^th^ month: 4 times/week; 8^th^ -16^th^ month: 3 times/week
Engsberg et al.^4^	--	--	8 months: 4 times/week + 12 months: 3 times/week
Schie et al.^16^	1^st^	1 week	5^th^ day: sitting on WC and therapy 3 times/day (1 hour); 6^th^ day: orthostasis and, when possible, gait with GRO; 3 months: 5 times/week (1 hour); 3^rd^-6^th^ month: 4 times/week (1 hour); 6^th^ -12^th^ month: 3 times/week (30 minutes)
Engsberg et al.^17^	3^rd^	1 week	1^st^ week: twice/day + 8 months: 4-5 times/week; after 8^th^ month: 3-4 times/week
Buckon et al.^21^	4^th^	1 month	1^st^ month: twice/day + occupational therapy: 1time/day; 2^th^ -6^th^ month: 3-4 times/week, occupational therapy: 1-2 times/ week; 6^th^ mês-1 year: 1-2 times/week
Steinbok et al.^13^	--	--	3 months: 3 times/week + 6 months: twice/week (9 months pre- and post-operative periods)
Engsberg et al.^15^	3^rd^	--	Post-operative period, 6 months: twice/week; 3^rd^ day post-operative period: 3 times/day; up to 6 months: 4-5 times/week; 6^th^ -8^th^ month: 3-4 times/week
Hodgkinson et al.^19^	--	--	6 months: once/day
Dudgeon et al.^20^	--	4 weeks	4 weeks: 2 hours/day, 5 times/week; occupational therapy: 3-5 hours/week + 5 months: 4-5 hours/week
Josenby et al.^23^	1^st^	--	6 months: 1 hour/2 times/week; up to 18 months: once/week and physical activities.
Nordmark et al.^5^	5^th^	3-5 days ICU	1^st^ week: 45 minutes/twice/day; 2^nd^ -3^rd^ week: 45 minutes/3 times/day; 2^nd^-6^th^ month: 1 hour/twice/week; 6 months: 1 hour/once/week
Steinbok et al.^8^	2^nd^	6 days	6^th^ day: weight support while standing up; 2^nd^ week: gait; 3 months: 3 times/week + 6 months: 2 times/week

PT: physical therapy; WC: wheelchair; GRO: ground-reaction orthosis. ICU: intensive care unit.

Half of the studies report that after the in-hospital physical therapy period, specific treatment guidelines are sent to local therapists, with whom the responsible therapist had made prior contact, in order to maintain consistency of the treatment plan.

As for the physical therapy program itself, early mobilization of the lower limbs is made during the first week after SDR to maintain a range of motion and positioning, including prone, supine and siting positions with extended lower limbs.^5^ ^,^ ^7^ ^,^ ^12^ ^,^ ^16^ The first five days are specific for muscle strength exercises with hip abductors and extensors, knee extensors, dorsiflexors, and practice of normal orthostasis and gait patterns are initiated.^16^ The onset of orthostasis is described as initiated by the use of parapodium in the 8^th^ day,^12^ or with the use of fixed or ground-reaction Ankle Foot Orthoses (AFO) to stimulate knee extension on the sixth day,^16^ and adaptation equipment.^14^ Muscle strengthening is described as rehabilitation practice in most studies,^4^ ^,^ ^7^ ^,^ ^8^ ^,^ ^11^ ^,^ ^12^ ^,^ ^13^ ^,^ ^14^ ^,^ ^16^ ^,^ ^17^ ^,^ ^20^ ^,^ ^21^ with emphasis on the lower limb extensor and hip abductors muscles, knee extensors and dorsiflexors,^8^ ^,^ ^12^ ^,^ ^13^ ^,^ ^16^ in addition to upper limbs^12^ and trunk muscle.,^4^ ^,^ ^12^ The exercises are performed using isolated training,^20^ progressive resistance training,^11^ and selective or functional control.^21^ Gait training starts on the second^7^ or third week^12^ and focuses on normal motor pattern with the use of supportive devices^17^ if necessary. In addition, the use of normal movement pattern facilitation (neurodevelopmental theory) is also described,^8^ ^,^ ^11^ ^,^ ^12^ ^,^ ^13^ ^,^ ^21^ as well as fine motor skills training,^12^ functional activities,^4^ ^,^ ^5^ ^,^ ^7^ ^,^ ^12^ ^,^ ^14^ ^,^ ^17^ ^,^ ^20^ ^,^ ^21^ ^,^ ^22^ daily-living activities,^5^ ^.^ ^7^ posture control and alignment,^8^ ^,^ ^13^ ^,^ ^14^ ^,^ ^22^ and postural transfer training with emphasis to balance when siting, kneeling, crawling, standing from floor and chair, standing, and on gait.^5^ ^,^ ^7^ ^,^ ^12^ ^,^ ^17^ ^,^ ^21^ ^,^ ^22^ Hydrotherapy,^5^ ^.^ ^16^ equotherapy,^5^ ^,^ ^16^ and physical activities^5^ ^,^ ^22^ ^,^ ^23^ are also mentioned.

According to the most recent recommendations by the National Institute for Health and Clinical Excellence (NICE), when it comes to treatment of spasticity in children and adolescents with non-progressive brain disorders, an intensive physical therapy program is essential after clinical approach to spasticity by SDR^31^ and also determinant for successful outcomes.^30^

## FINAL REMARKS

Several studies have reported the treatment of spasticity by SDR associated with physical therapy. At large, they emphasize the importance of adequate indication of the procedure to be made by a multidisciplinary team that includes a physical therapist. The most important indication is for outpatients presenting spastic diplegia, as a means to improve gait and motor function patterns. A less frequent indication is for patients with spastic quadriplegia, with specific goals of positioning, spasticity control, sitting, hygiene, and daily care for both patient and relatives. Intensive and long-term postoperative physical therapy (especially in the first postoperative year) is emphasized and should cover strategies to modify the patient’s former motor patterns.

Further prospective studies with long-term follow-up rehabilitation protocols are suggested. The use of validated evaluation instruments for the analysis of both static/functional aspects and quality of life should be considered, aiming to clarify SDR indication criteria and whether the current postoperative rehabilitation conventions are appropriate.

Thus, this literature review shows that physical therapy plays a key role in the rehabilitation of patients with CP who was submitted to SDR. Such role takes place from the initial selection of patients - along with the team -, pre- and postoperative evaluations, through rehabilitation. This review may assist health professionals in the post-SDR treatment of patients with bilateral spastic CP.

## References

[1] McLaughlin J, Bjornson K, Temkin N, Steinbok P, Wright V, Reiner A (2002). Selective dorsal rhizotomy: meta-analysis of three randomized
controlled trials. Dev Med Child Neurol.

[2] Aquilina K, Graham D, Wimalasundera N (2015). Selective dorsal rhizotomy: an old treatment
re-emerging. Arch Dis Child.

[3] Grunt S, Fieggen AG, Vermeulen RJ, Becher JG, Langerak NG (2014). Selection criteria for selective dorsal rhizotomy in children with
spastic cerebral palsy: a systematic review of the literature. Dev Med Child Neurol.

[4] Engsberg JR, Ross SA, Collins DR, Park TS (2006). Effect of selective dorsal rhizotomy in the treatment of children with
cerebral palsy. J Neurosurg.

[5] Nordmark E, Josenby AL, Lagergren J, Andersson G, Stromblad LG, Westbom L (2008). Long-term outcomes five years after selective dorsal
rhizotomy. BMC Pediatr.

[6] Graubert C, Song KM, McLaughlin JF, Bjornson KF (2000). Changes in gait at 1 year post-selective dorsal rhizotomy: results of
a prospective randomized study. J Pediatr Orthop.

[7] Chan SH, Yam KY, Yiu-Lau BP, Poon CY, Chan NN, Cheung HM (2008). Selective dorsal rhizotomy in Hong Kong: multidimensional outcome
measures. Pediatr Neurol.

[8] Steinbok P, Reiner AM, Beauchamp R, Armstrong RW, Cochrane DD, Kestle J (1997). A randomized clinical trial to compare selective posterior rhizotomy
plus physiotherapy with physiotherapy alone in children with spastic diplegic
cerebral palsy. Dev Med Child Neurol.

[9] Steinbok P (2007). Selective dorsal rhizotomy for spastic cerebral palsy: a
review. Childs Nerv Syst.

[10] Hendricks-Ferguson VL, Ortman MR (1995). Selective dorsal rhizotomy to decrease spasticity in cerebral
palsy. AORN J.

[11] McLaughlin JF, Bjornson KF, Astley SJ, Graubert C, Hays RM, Roberts TS (1998). Selective dorsal rhizotomy: efficacy and safety in an
investigator-masked randomized clinical trial. Dev Med Child Neurol.

[12] Wright FV, Sheil EM, Drake JM, Wedge JH, Naumann S (1998). Evaluation of selective dorsal rhizotomy for the reduction of
spasticity in cerebral palsy: a randomized controlled tria. Dev Med Child Neurol.

[13] Steinbok P, McLeod K (2002). Comparison of motor outcomes after selective dorsal rhizotomy with and
without preoperative intensified physiotherapy in children with spastic diplegic
cerebral palsy. Pediatr neurosurg.

[14] McLaughlin JF, Bjornson KF, Astley SJ, Hays RM, Hoffinger SA, Armantrout EA (1994). The role of selective dorsal rhizotomy in cerebral palsy: critical
evaluation of a prospective clinical series. Dev Med Child Neurol.

[15] Engsberg JR, Olree KS, Ross SA, Park TS (1998). Spasticity and strength changes as a function of selective dorsal
rhizotomy. Neurosurg focus.

[16] Schie PE, Vermeulen RJ, Ouwerkerk WJ, Kwakkel G, Becher JG (2005). Selective dorsal rhizotomy in cerebral palsy to improve functional
abilities: evaluation of criteria for selection. Childs Nerv Syst.

[17] Engsberg JR, Ross SA, Wagner JM, Park TS (2002). Changes in hip spasticity and strength following selective dorsal
rhizotomy and physical therapy for spastic cerebral palsy. Dev Med Child Neurol.

[18] Engsberg JR, Ross SA, Collins DR, Park TS (2007). Predicting functional change from preintervention measures in
selective dorsal rhizotomy. J Neurosurg.

[19] Hodgkinson I, Berard C, Jindrich ML, Sindou M, Mertens P, Berard J (1997). Selective dorsal rhizotomy in children with cerebral palsy. Results in
18 cases at one year postoperatively. Stereotact Funct Neurosurg.

[20] Dudgeon BJ, Libby AK, McLaughlin JF, Hays RM, Bjornson KF, Roberts TS (1994). Prospective measurement of functional changes after selective dorsal
rhizotomy. Arch Phys Med Rehabil.

[21] Buckon CE, Thomas SS, Harris GE, Piatt JH, Aiona MD, Sussman MD (2002). Objective measurement of muscle strength in children with spastic
diplegia after selective dorsal rhizotomy. Arch Phys Med Rehabil.

[22] Josenby AL, Wagner P, Jarnlo GB, Westbom L, Nordmark E (2012). Motor function after selective dorsal rhizotomy: a 10-year
practice-based follow-up study. Dev Med Child Neurol.

[23] Josenby AL, Wagner P, Jarnlo GB, Westbom L, Nordmark E (2015). Functional performance in self-care and mobility after selective
dorsal rhizotomy: a 10-year practice-based follow-up study. Dev Med Child Neurol.

[24] Gump WC, Mutchnick IS, Moriarty TM (2013). Selective dorsal rhizotomy for spasticity not associated with cerebral
palsy: reconsideration of surgical inclusion criteria. Neurosurg Focus.

[25] Mazarakis NK, Vloeberghs MH (2016). Spasticity secondary to Leigh syndrome managed with selective dorsal
rhizotomy: a case report. Childs Nerv Syst.

[26] Eppinger MA, Berman CM, Mazzola CA (2015). Selective dorsal rhizotomy for spastic diplegia secondary to stroke in
an adult patient. Surg Neurol Int.

[27] Mazarakis NK, Ughratdar I, Vloeberghs MH (2015). Excellent functional outcome following selective dorsal rhizotomy in a
child with spasticity secondary to transverse myelitis. Childs Nerv Syst.

[28] Ingale H, Ughratdar I, Muquit S, Moussa AA, Vloeberghs MH (2016). Selective dorsal rhizotomy as an alternative to intrathecal baclofen
pump replacement in GMFCS grades 4 and 5 children. Childs Nerv Syst.

[29] Reynolds MR, Ray WZ, Strom RG, Blackburn SL, Lee A, Park TS (2011). Clinical outcomes after selective dorsal rhizotomy in an adult
population. World Neurosurg.

[30] Giuliani CA (1991). Dorsal rhizotomy for children with cerebral palsy: support for
concepts of motor control. Phys Ther.

[31] Mugglestone MA, Eunson P, Murphy MS, Guideline Development Groop (2012). Spasticity in children and young people with non-progressive brain
disorders: summary of NICE guidance. BMJ.

